# Autophagosome accumulation-mediated ATP energy deprivation induced by penfluridol triggers nonapoptotic cell death of lung cancer via activating unfolded protein response

**DOI:** 10.1038/s41419-019-1785-9

**Published:** 2019-07-15

**Authors:** Wen-Yueh Hung, Jer-Hwa Chang, Yu Cheng, Guo-Zhou Cheng, Hsiang-Ching Huang, Michael Hsiao, Chi-Li Chung, Wei-Jiunn Lee, Ming-Hsien Chien

**Affiliations:** 1 Graduate Institute of Clinical Medicine, College of Medicine, Taipei Medical University, Taipei, Taiwan; 2 Division of Pulmonary Medicine, Department of Internal Medicine, School of Medicine, College of Medicine, Taipei Medical University, Taipei, Taiwan; 3 Division of Pulmonary Medicine, Department of Internal Medicine, Wan Fang Hospital, Taipei Medical University, Taipei, Taiwan; 4 Pulmonary Research Center, Wan Fang Hospital, Taipei Medical University, Taipei, Taiwan; 5 Graduate Institute of Medical Sciences, College of Medicine, Taipei Medical University, Taipei, Taiwan; 6 Genomics Research Center, Academia Sinica, Taipei, Taiwan; 7 Division of Pulmonary Medicine, Department of Internal Medicine, Taipei Medical University Hospital, Taipei, Taiwan; 8 Department of Medical Education and Research, Wan Fang Hospital, Taipei Medical University, Taipei, Taiwan; 9 Department of Urology, School of Medicine, Taipei Medical University, Taipei, Taiwan; 10 Cancer Center, Wan Fang Hospital, Taipei Medical University, Taipei, Taiwan; 11 TMU Research Center of Cancer Translational Medicine, Taipei Medical University, Taipei, Taiwan

**Keywords:** Drug development, Non-small-cell lung cancer

## Abstract

Anticancer chemotherapeutic drugs mainly trigger apoptosis induction to eliminate malignant cells. However, many cancer cells are chemoresistant because of defective apoptosis induction. Targeting the autophagic pathway is currently regarded as an alternative strategy for cancer drug discovery. Penfluridol, an antipsychotic drug, has been reported to exert oncostatic effects, but the effect of penfluridol on lung cancer remains unknown. Herein, the antitumor activity of penfluridol was determined in vitro in non-small-cell lung cancer (NSCLC) cell lines using MTS, plate clonogenic, and transwell migration assays and in vivo in an orthotopic xenograft model. Flow cytometry, holotomographic microscopy, immunofluorescence, and immunohistochemistry were employed to determine the cell-death phenotype induced by penfluridol in vitro and in vivo. Western blotting and genetic knockdown by small interfering RNA were performed to explore the underlying mechanisms involved in penfluridol-mediated cell death. We uncovered that penfluridol inhibited the viability and motility of NSCLC cells in vitro and in vivo. Penfluridol induced nonapoptotic cell death by blocking autophagic flux and inducing accumulation of autophagosome-related protein, light chain 3 (LC3) B-II, in HCC827 and A549 NSCLC cells, and in an A549 orthotopic xenograft tumor model. Autophagosome accumulation-induced cell viability inhibition by penfluridol was mainly attributed to ATP energy deprivation. Moreover, we observed that patients with lung tumors expressing high LC3B had longer overall and disease-free survival times. Mechanistically, upregulation of endoplasmic reticulum (ER) stress-induced unfolded protein response (UPR) pathways and activation of p38 mitogen-activated protein kinase (MAPK) were critical for penfluridol-induced autophagosome accumulation. Our findings identify that penfluridol acts as an inducer of ER stress and p38 MAPK activation, which led to UPR-mediated nonapoptotic cell death via autophagosome accumulation-caused energy loss. Penfluridol is clinically used for schizophrenia, and our study results strongly support penfluridol as a repurposed drug for treating NSCLC.

## Introduction

Lung cancer is one of the major causes of cancer-related deaths worldwide^1^, and the main lung cancer type is non-small-cell lung cancers (NSCLCs). Traditional treatments for NSCLCs are platinum- and cisplatin-based chemotherapies^2^, but treatment failure is frequently observed. Lung cancers are typically resistant to apoptosis induced by chemotherapy due to molecular modifications such as induction of antiapoptotic genes or mutations in intrinsic apoptotic signaling^3^. For example, high expression levels of the antiapoptotic proteins Bcl-2 and Bcl-xL were reported to be correlated with cisplatin resistance and tumor recurrence in NSCLCs^4^. Hence, the ability to engage nonapoptotic cell death might provide an alternative strategy for NSCLC treatment.

Besides apoptosis, many other cell death forms have recently been identified such as autophagic cell death. Activation of autophagy is closely related to tumor cell sensitivity to anticancer drugs and radiation^5^. In established tumors, autophagy was reported to act as a prosurvival pathway, which favors tumor progression and mediates drug resistance. However, persistent and excessive induction of autophagy by anticancer agents can induce irreversible destruction of cellular contents and eventually lead to cell death^6^. Recently, induction of autophagosome accumulation, which cooperates with defective lysosomal activity, was reported to induce cell death, including cancer cells^7,8^. This suggests that autophagy might have differential impacts on distinct phases and different types of cancer development. As to the role of autophagy in lung cancer, haploinsufficiency of a key autophagy mediator, *Beclin1*, in genetically modified mice resulted in the formation of lung adenocarcinomas, and the Beclin-1 expression level was positively correlated with overall survival of patients with NSCLCs^9,10^. To date, various pharmacological agents such as natural compounds or small molecules were shown to induce autophagic cell death in several cancer types including NSCLCs^11,12^. Therefore, activation of autophagy in apoptosis-resistant cancers could potentially provide a way to induce cell death and retard tumor growth.

The endoplasmic reticulum (ER) serves as the intracellular protein-folding factory. The accumulation of unfolded proteins in the ER leads to stress conditions referred to as ER stress. Next, stressed cells turn on a homeostatic intracellular signaling network cumulatively called the unfolded protein response (UPR), which helps recuperate ER function^13^. ER stress is reported to induce autophagy and share several common features with autophagy such as protecting cells by relieving stress and inducing cell death under extreme conditions, and this phenomenon can also be observed in cancer cells in response to anticancer drugs^14^. The roles of ER stress and autophagy have been established in cancer progression and chemoresistance including in lung cancer^15^. The ER stress-related marker, GRP78, and Beclin show the significant correlation with longer overall survival in invasive NSCLCs, suggesting that ER-induced autophagy is a druggable target for NSCLC treatment^16^.

Although many natural compounds have been reported to induce autophagic or other nonapoptotic cell death in various cancers^11^, the major limitations of such compounds for clinical use are their low oral bioavailability^17^. Developing new uses of a drug in addition to its initial approved indication, also known as drug repurposing, is a promising strategy in translational medicine for obtaining more therapeutic drugs. Repurposing can help identify new therapies for diseases at lower cost and in a shorter time, particularly in those cases where preclinical safety profiles have already been identified and newly identified use(s) can be rapidly evaluated in clinical trials^18^. For example, nonsteroidal anti-inflammatory drugs and antidiabetic drugs are all successful cases of repurposing^19–21^.

Penfluridol is a long-acting oral antipsychotic drug for treating schizophrenia^22^. Recently, penfluridol was shown to be a repurposed drug for cancer treatment such as inhibiting growth and metastasis in several cancer types including breast, pancreatic, and brain tumors^23–29^. Multiple mechanisms such as increasing reactive oxygen species (ROS), downregulating Sp transcription factors, inhibiting integrin signaling, activating protein phosphatase 2A, modulating immune responses, and enhancing radiosensitivity are reported to be involved in the anticancer activities of penfluridol on various tumor types^23–29^. In contrast to those tumor types, the anticancer potential and the underlying mechanisms of penfluridol on NSCLCs are still unknown.

In the present study, we investigated the anticancer effects of penfluridol on NSCLC cell lines (A549 and HCC827), which harbor the wild-type or mutant epidermal growth factor receptor (EGFR) and its underlying mechanisms in vitro and in an orthotopic xenograft model. We found that penfluridol at a nontoxic concentration suppressed the motility of NSCLC cells. Moreover, penfluridol exerted cytotoxic effects on NSCLC cells in vitro and in vivo by inducing nonapoptotic cell death with features of autophagosome accumulation. Autophagosome accumulation-mediated cell growth inhibition by penfluridol was mainly attributed to adenosine triphosphate (ATP) energy deprivation. The ER stress-induced p38-mediated UPR is a major mechanism involved in penfluridol-induced death of NSCLC cells via autophagosome accumulation. Based on these observations, manipulation of autophagy might be a feasible NSCLC treatment with penfluridol in future clinical applications.

## Materials and methods

### Reagents, antibodies, and commercial kits

Penfluridol (P3371), dimethyl sulfoxide (DMSO), 3-methylamphetamine (3-MA), chloroquine (CQ), glutathione (GSH), N-acetylcysteine (NAC), acridine orange (AO), 2′,7′-dichlorofluorescin diacetate (DCF-DA), ATP, rapamycin, and zVAD-fmk were all purchased from Sigma-Aldrich (St. Louis, MO). Propidium iodide (PI), fetal bovine serum (FBS), antibiotics, trypsin-EDTA, trypan blue stain, and all medium additives were obtained from Life Technologies (Gaithersburg, MD). The p38 inhibitor, SB203580, was purchased from Calbiochem (San Diego, CA). An enhanced chemiluminescence (ECL) kit was purchased from Amersham (Arlington Heights, IL). Antibodies, specifically of caspase-3, poly(ADP ribose) polymerase (PARP), and unphosphorylated or phosphorylated (p-) forms of the corresponding extracellular signal-regulated kinase 1/2 (ERK1/2), p38, and c-Jun N-terminal kinase 1/2 (JNK1/2) were all purchased from Cell Signaling Technology (Danvers, MA). Antibodies specific for UPR pathways including GRP94, GRP78, ERO1α, PERK, IRE1α, CHOP, and p-eIF2α, and autophagy-related proteins including LC3B, ATG5, and p62 were also purchased from Cell Signaling Technology. Antibodies specific for Beclin-1 p21, p27, specificity protein (Sp) 1, Sp3, Sp4, and β-actin were obtained from Santa Cruz Biotechnology (Santa Cruz, CA). Polyvinylidene fluoride membranes for western blotting were purchased from Bio-Rad (Hercules, CA). Unless otherwise specified, other chemicals used in this study were purchased from Sigma Chemical (St. Louis, MO).

### Cell lines and cell cultures

The human NSCLC A549 and HCC827 cell lines and human bronchial/lung epithelial BEAS-2B cells were purchased from the American Type Culture Collection (Manassas, VA) and were kept frozen until initiation of these studies. NSCLC cells and BEAS-2B cells were respectively maintained in RPMI 1640 and Dulbecco’s modified Eagle’s medium supplemented with 10% FBS and 1% penicillin–streptomycin–glutamine. All cells were incubated at 37 °C in a humidified 5% CO_2_ atmosphere.

### Cell viability assay (MTS assay)

Cells were plated at a density of 5000 cells/well in 96-well plates and incubated overnight. Cells were next treated with various concentrations of penfluridol (1.25–40 μM) for 24, 48, and 72 h, and then subjected to a cell-viability assay (MTS assay; Promega, Madison WI) according to the manufacturer’s instructions. Data were collected from three replicates.

### Plate clonogenic assay

NSCLC cells were seeded at 1000 cells/well of a six-well plate and incubated for 24 h. Cells were next treated with various concentrations (1.25–20 μM) of penfluridol for 24 h, and then continuously incubated in new fresh medium at 37 °C. After incubation for 7–10 days, cells were stained with crystal violet, and colonies were counted manually using ImageJ free software (NIH, Bethesda, MD).

### Transwell cell migration assay

A549 and HCC827 cells (3 × 10^4^) were plated in a noncoated top chamber (24-well insert; pore size, 8 μm; Corning Costar, Corning, NY) for the transwell migration assay. Cells pretreated for 24 h with penfluridol (1.25–20 μM) were plated in medium without serum or growth factors, and medium supplemented with 10% serum was used as a chemoattractant in the lower chamber. After another 24 h, cells, which passed through the membrane, were fixed with methanol and stained with crystal violet. The number of cells migrating through the membrane was counted under a light microscope (×100, three random fields per well).

### Fluorescence-activated cell sorting (FACS) analysis

After treatment of NSCLC cells with penfluridol (5 and 10 μM) for 24 h, A549 and HCC827cells (10^6^) were trypsinized from the dish and collected via centrifugation. Cells were then washed by phosphate-buffered saline (PBS), fixed with ice-cold 80% (v/v) ethanol at −20 °C for 12 h, and stained with (PI; 50 μg/mL) in the presence of RNase A (100 μg/mL). The DNA contents were measured using a FACScan laser flow cytometer analysis system (Beckman Coulter, Los Angeles, CA). The proportion of nuclei in each phase of the cell cycle was determined, and apoptotic cells with hypodiploid DNA content were detected in the sub-G_1_ region.

### Nuclear morphological analysis by DAPI

After treatment of both A549 and HCC827 cells with penfluridol for 24 h, the monolayer of cells was washed with PBS and fixed with methanol for 10 min at room temperature (RT). Cells were washed with PBS and incubated for 5 min with a DAPI solution, and examined and photographed using a Zeiss Axiophot fluorescence microscope (Carl Zeiss Microimaging, Gottingen, Germany). Apoptotic cells exhibited morphological features of apoptosis including chromatin condensation and nuclear fragmentation.

### Tomographic microscopy

A549 and HCC827 human ling cancer cells were seeded into 35-mm tissue culture dishes (FluoroDish, World Precision Instruments, Sarasota, FL) at 15,000 cells/dish and allowed to grow overnight. Cells were treated with 7.5 μM of penfluridol for 24 h or left untreated, and then cells were switched to phenol red-free medium. Dishes were then placed on a holotomographic microscope (3D Explorer, NanoLive, Lausanne, Switzerland), and images are taken at ×600 magnification.

### Autophagosome detection

To study the effect of penfluridol on autophagosome formation, 10^4^ cells were seeded onto sterile coverslips, cultured under standard conditions for 24 h, and treated with penfluridol, CQ, or rapamycin for another 18 h. Cells were then stained with or without LysoTracker Red for 30 min at 37 °C. After staining, cells were washed three times with PBS and fixed in 4% paraformaldehyde at RT followed by permeabilization with 0.1% Triton X-100 for 10 min. After washing, cells were blocked by incubation with 10% BSA for 30 min, followed by incubation with a primary anti-LC3 antibody (1:100) for 1 h. Then, cells were incubated with AlexaFluor488-conjugated anti-rabbit secondary antibody (Thermo Fisher Scientific, Rockford, IL) for 45 min at RT. The nuclei were counterstained with DAPI. After washing with PBS, the slides were examined and photographed using a Zeiss Axiophot fluorescence microscope (Carl Zeiss Microimaging) with an objective magnification of ×40 under identical conditions in each experiment.

### Autophagic vacuole determination by AO staining

Autophagy is the process of sequestering cytoplasmic proteins into a lytic compartment and is characterized by the formation and accumulation of acidic vesicular organelles (AVOs). To detect acidic cellular compartments, we used AO, which emits bright red fluorescence in acidic vesicles and green fluorescence in the cytoplasm and nucleus. Cells were seeded into 24-well plates and treated with 7.5 μM penfluridol for 6 h. Subsequently, AO was added to the medium at a final concentration of 1 μg/mL for 15 min, and cells were then washed with PBS. Images were captured using a Zeiss Axiophot fluorescence microscope (Carl Zeiss Microimaging). As an autophagy control, cells were serum-starved for 48 h.

### Small-interfering (siRNA) transfection

siRNA for human light chain 3 (LC3) (sc-43390) and a nonrelated control siRNA (sc-37007) were obtained from Santa Cruz Biotechnology. To knock down LC3, 70% confluent NSCLC cells in a 6-mm^2^ Petri dish were transfected with 50 or 100 nM of siRNA using GenMute™ siRNA Transfection Reagent (SignaGen Laboratories, Gaithersburg, MD) for 6 h according to the manufacturer’s instructions. At 24 h after transfection, cells were analyzed for proliferation and expression of LC3.

### Preparation of total cell extracts and western blot analysis

Protein lysates were prepared as described previously^30^. The protein content was determined with the Bio-Rad protein assay reagent using bovine serum albumin as a standard. A western blot analysis was performed with indicated primary antibodies and horseradish peroxidase-conjugated secondary antibodies. After washing, blots were incubated with the western blotting reagent ECL, and chemiluminescence was detected by the chemiluminescence imaging system, MultiGel-21, (TOP BIO, New Taipei City, Taiwan). Furthermore, the same blots were stripped with stripping buffer (TOOLS, New Taipei City, Taiwan) and reprobed with a β-actin or α-tubulin antibody as the internal control. Densitometric analysis of the bands was carried out using Image-Pro Plus software (Media Cybernetics Inc. Rockville, MD) to define the boundaries of protein bands, assess density, and to obtain final numerical data. The intensity of bands from the protein of interest was normalized to the intensity of actin or tubulin bands of the respective blots.

### In vivo antitumor activity of penfluridol treatment

Male nonobese diabetic (NOD)-SCID mice (4–6 weeks) were used in assays for tumor growth in an orthotopic graft model. All animal experiments were performed in accordance with guidelines of the Institutional Animal Care and Use Committee (IACUC) of Taipei Medical University. Mice were anesthetized with isoflurane and placed in the right lateral decubitus position; then 10^6^ luciferase-tagged A549 cells (A549-Luc) were suspended in a 1:1 mixture of PBS and growth factor-reduced (GFR) Matrigel and injected into the left lung parenchyma of NOD-SCID mice. After 7 days, mice were randomized into three groups according to bioluminescence images taken with the Xenogen IVIS-Spectrum system (Caliper; Xenogen), and treatment was initiated according to similar mean tumor sizes in each group. Subsequently, the mice were orally given 5 or 10 mg/kg penfluridol or the vehicle (10% DMSO in PBS) 5 days per week. The following day after penfluridol treatment, the mice were injected with d-luciferin and imaged for 1–5 min using this live imaging device to monitor the tumor size and location in real time. Thirty-five days after penfluridol treatment, the mice were killed, tumor specimens were resected, and immunohistochemical (IHC) staining or western blotting was performed.

### IHC

All tumor tissue samples were fixed in a buffered 10% formaldehyde solution. Fixed tumor tissues were dehydrated and embedded in paraffin blocks and cut into 4-μm sections. All specimens were deparaffinized with xylene, ethanol, and double-distilled water washes and immersed in 10 mM sodium citrate buffer (pH 6.0) in a microwave oven twice for 5 min to enhance antigen retrieval. After washing, slides were incubated with 0.3% hydrogen peroxide in methanol to quench endogenous peroxidase activity. Slides were washed with PBS and incubated with an anti-LC3B antibody for 2 h at RT. After washing in PBS, slides were developed with a VECTASTAIN ABC peroxidase kit (Vector Laboratories, Burlingame, CA) and DAB peroxidase substrate kit (Vector Laboratories) according to the manufacturer’s instructions. All specimens were deparaffinized and stained with hematoxylin and eosin, which was used as a light counterstain.

### Measurement of the intracellular ROS levels

Intracellular ROS production for NSCLC cells treated with penfluridol was measured using the fluoroprobe DCF-DA. After treatment of cells with penfluridol for 6 h, the cells were incubated in the dark in 50 μM DCF-DA for 30 min at 37 °C. Images were taken using a fluorescence microscope (Zeiss Axioplan). To quantify the intensity of DCF fluorescence, flow cytometry was used.

### Analysis of intracellular ATP levels

Cells were plated in 96-well plates at a density of 5000 cells/well. After 20–24 h of incubation, cells were next treated with various concentrations of penfluridol (2.5–7.5 μM) for 24 h, and then subjected to detect the intracellular ATP by a Luminescent ATP Detection Assay Kit (Abcam, Cambridge, MA) according to the manufacturer’s instructions. The luminescence was normalized to the number of viable cells.

### Statistical analysis

Values are presented as the mean ± standard deviation. Statistical analyses were performed using the Statistical Package for Social Science software, vers. 16 (SPSS, Chicago, IL). Data were analyzed using Student’s *t*-test with *p* < 0.05 as the criterion of significance when two groups were compared.

## Results

### In vitro anticancer activities of penfluridol at toxic and nontoxic concentrations in human NSCLC cells

**Fig. 1:**
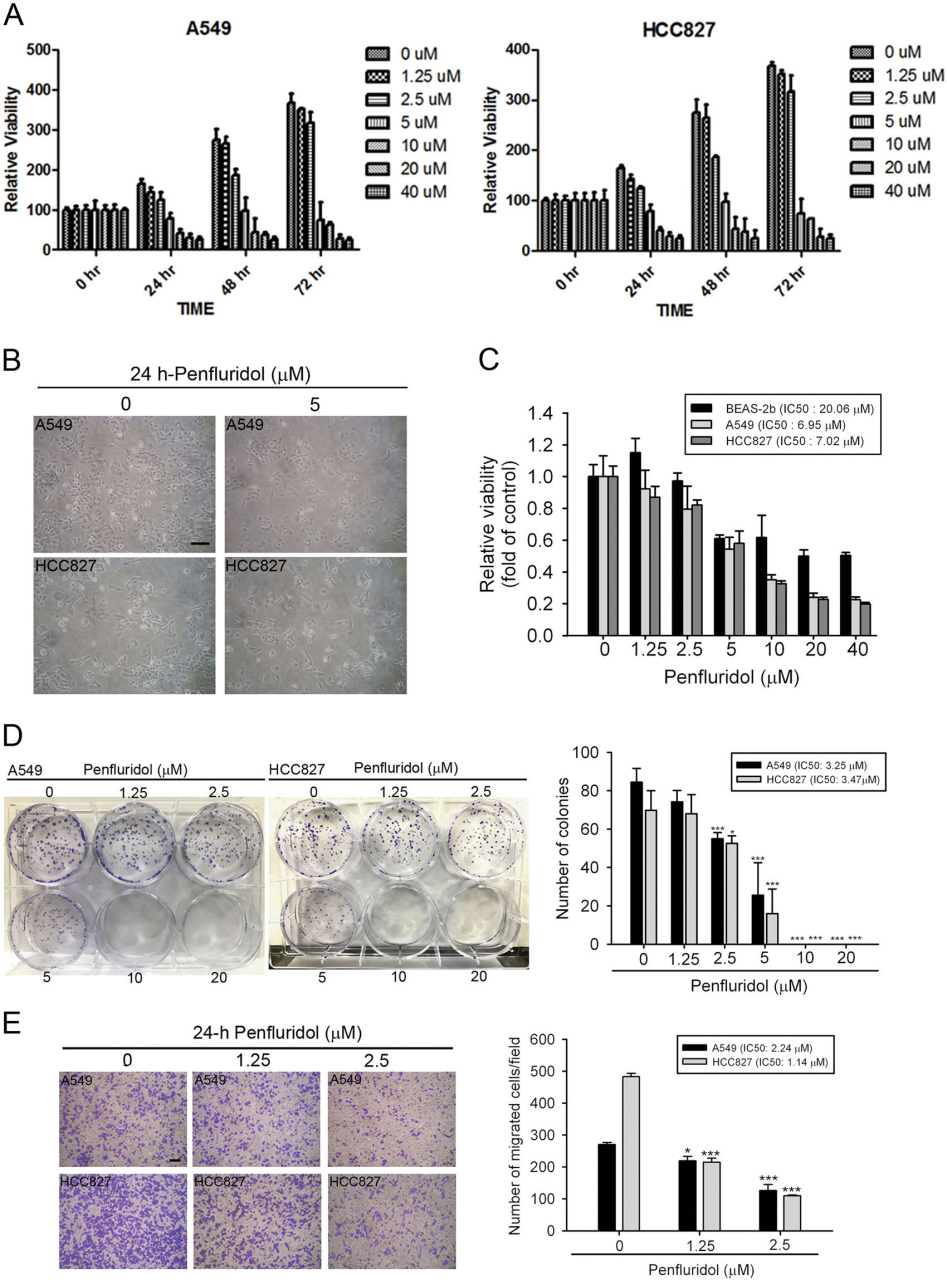
Penfluridol suppresses the proliferation, colony formation, and motility of human non-small-cell lung cancer (NSCLC) cells. **a** Two NSCLC cell lines, A549 (wild-type EGFR) and HCC827 (mutant EGFR, del E746-A750), were treated with different concentrations of penfluridol for 24, 48, or 72 h, and cell viability was determined with an MTS assay. **b** A549 or HCC827 cells were treated with the vehicle or penfluridol (5 μM) for 24 h; then, the cell morphology was photographed using a phase-contrast microscope (×100). **c** NSCLC cells and normal lung epithelial cells (BEAS-2b) were treated with the indicated concentrations of penfluridol for 24 h, and the half maximal inhibitory concentration (IC_50_) of these cells was determined by an MTS assay. **d** A549 and HCC827 cells were treated with the vehicle or penfluridol (1.25–20 μM) for 24 h; then, the death-inducing effects of penfluridol on cells were determined by counting the colonies formed. **e** A549 and HCC827 cells were treated with 1.25 or 2.5 μM of penfluridol for a transwell migration assay. Left panels of **d**, **e**: representative photomicrographs. Right panels of **d**, **e**: values are presented as the mean ± SD from three independent experiments. *p *<* 0.05, ***p < 0.001 compared to the vehicle group

To investigate the pharmacological potential of penfluridol against NSCLC, we first examined the short-term treatment (24–72 h) effect of penfluridol on cell proliferation in NSCLC cells using an MTS assay. The cytotoxic effects of penfluridol treatment at indicated time points and various concentrations (1.25–40 μM) on two NSCLC cell lines (A549 and HCC827) are shown in Fig. 1a. Values of the half maximal inhibitory concentration (IC_50_) of penfluridol were 6.9, 5.8, and 4.8 μM in A549 cells following 24, 48, and 72 h of treatment respectively. Similar to A549 cells, IC_50_ values were respectively 7, 5.2, and 4 μM in HCC827 cells following 24, 48, and 72 h of penfluridol treatment (Fig. 1a). Morphological changes in NSCLC cells were visualized via optical microscopy after cells were treated with penfluridol (5 μM) for 24 h. Cell densities of A549 and HCC827 cells dramatically decreased, but no characteristics of cell apoptosis were evident, such as the appearance of cell shrinkage in the penfluridol-treated group (Fig. 1b). In contrast to NSCLC cells, the IC_50_ of penfluridol in normal BEAS-2b lung epithelial cells was much higher than in NSCLC cells with 24 h of treatment (Fig. 1c). In addition to short-term treatment, a colony formation assay showed that long-term treatment (7–10 days) with penfluridol significantly inhibited the colony-forming abilities of A549 and HCC827 cells (Fig. 1d). Taken together, these results suggest potential cytotoxic effects of penfluridol specific to NSCLC cells rather than to normal lung epithelial cells. To further determine the effects of penfluridol on cell motility of NSCLC cells, a transwell migration assay was performed, and we found that penfluridol significantly inhibited migration of NSCLC cells at non- or low-cytotoxic concentrations (1.25–2.5 μM) (Fig. 1e).

### Penfluridol-mediated growth inhibition of human NSCLC cells is independent of apoptosis

**Fig. 2:**
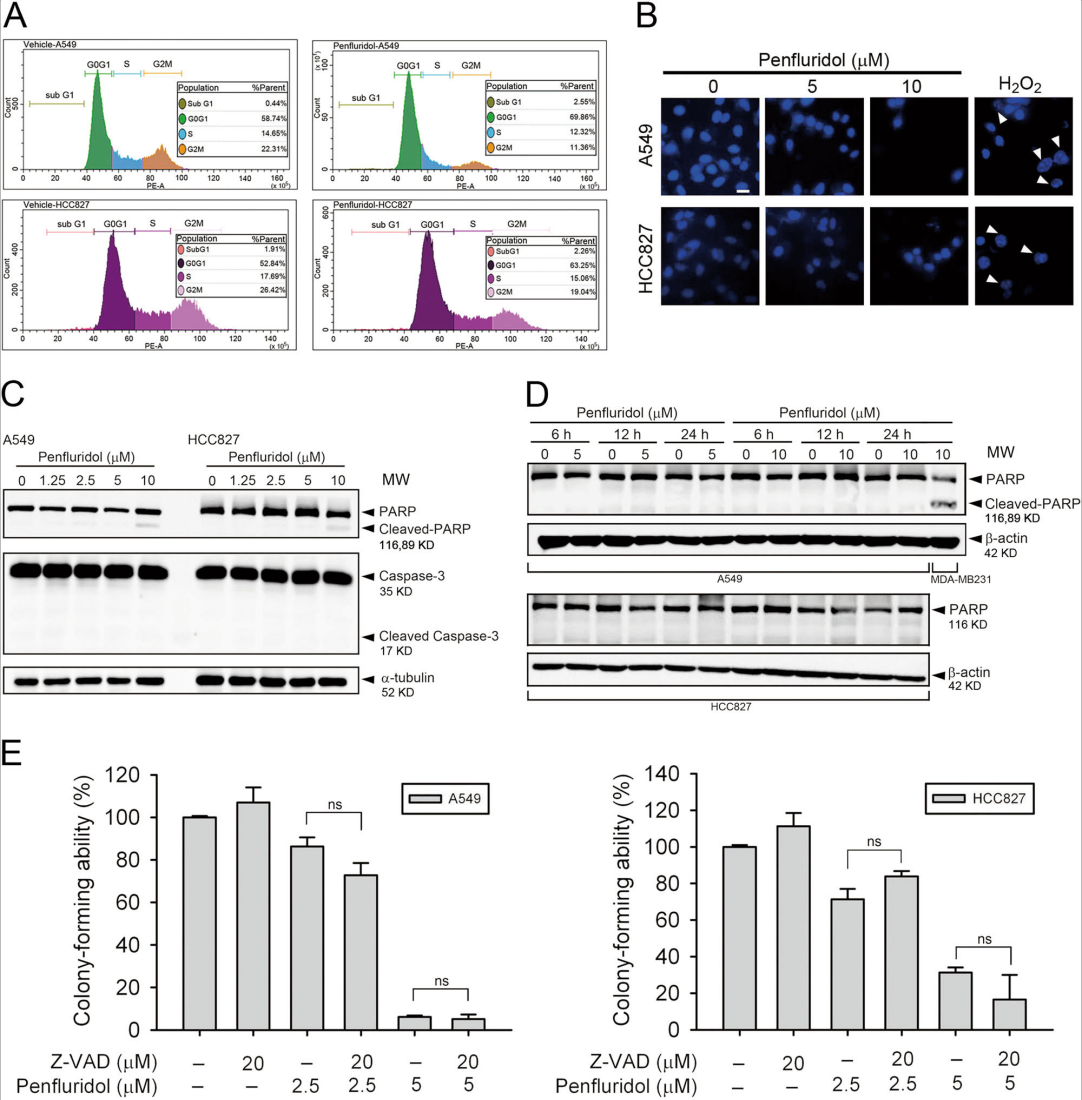
Penfluridol induces G_0_/G_1_ phase arrest and nonapoptotic cell death in non-small-cell lung cancer (NSCLC) cells. **a** Treatment of A549 and HCC827 cells with penfluridol (10 µM) for 24 h. The cell-cycle phase distribution and cell death in the sub-G_1_ phase were analyzed by FACS after propidium iodide (PI) staining. Data are shown as the cell-cycle distribution profile by FACS and the percentage distribution of cells in the sub-G_1_, G_0_/G_1_, S, and G_2_/M phases are inserted in the graphs. **b** A549 and HCC827 cells were treated with 100 µM H_2_O_2_ or indicated concentrations of penfluridol for 24 h, apoptotic nuclear fragmentation (arrows) as an indicator of apoptosis was analyzed by fluorescence microscopy after DAPI staining. Expression of cleaved caspase-3 and poly(ADP ribose) polymerase (PARP) were evaluated by a western blot analysis after treatment of A549 and HCC827 cells with various concentrations of penfluridol for 24 h (**c**) or 5 or 10 µM penfluridol for different time points (6, 12, 24 h) (**d**). MDA-MB231 cells were used as a positive control for penfluridol-induced PARP cleavage. **e** A549 and HCC827 cells were pretreated with and without 20 µM Z-VAD-FMK for 1 h followed by penfluridol (2.5 or 5 μM) treatment for an additional 24 h. The death-inducing effects of penfluridol on cells were determined by counting the colonies formed. Data are presented as the mean ± SD of three independent experiments. ns, nonsignificant *p* value

Previous reports indicated that apoptosis induction is the major cause of the growth-suppressive effects of penfluridol on cancers^26–28^. Herein, A549 and HCC827 cells were treated with 5 and 10 μM of penfluridol for 24 h and the flow cytometric cell cycle analysis showed a concentration-dependent increase in the accumulation of cell populations in the G_0_/G_1_ phase, but not in the sub-G_1_ phase (Fig. 2a and S1). No substantial apoptosis-related morphological changes such as condensed nuclei were observed in A549 or HCC827 cells. H_2_O_2_ was used as a positive control for apoptosis induction in these two cell lines (Fig. 2b). Moreover, the apoptotic markers, cleaved caspase-3 and PARP, were not significantly induced after treatment with different concentrations of penfluridol (1.25–10 μM) for 24 h (Fig. 2c) or 5 and 10 μM penfluridol treatment for different time points (6, 12, and 24 h) (Fig. 2d). The MDA-MB231 breast cancer cell line was used as a positive control for the penfluridol-induced increase in PARP cleavage (Fig. 2d). Under tomographic microscopy, we observed that 24 h of treatment of penfluridol (7.5 μM) did not induce the formation of apoptotic bodies in A549 or HCC827 cells (Fig. S2). Furthermore, Fig. 2e shows that the penfluridol-induced decreases in colony formation were not significantly affected by pretreatment with the apoptosis inhibitor, Z-VAD. Overall, these data indicated that the penfluridol-induced viability inhibition of A549 and HCC827 cells does not involve the apoptotic cell death pathway.

### Penfluridol induces autophagosome formation in NSCLC cells

**Fig. 3:**
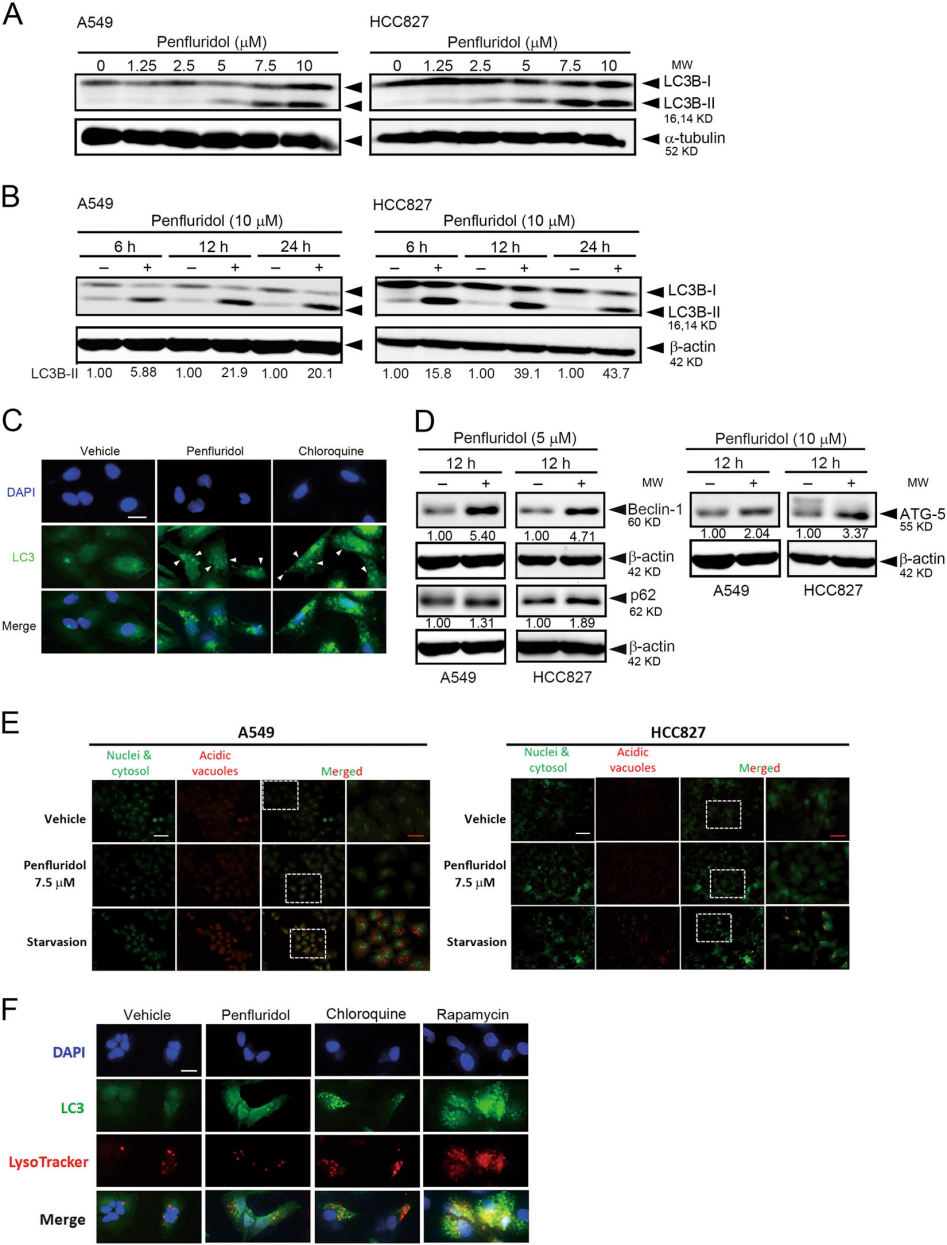
Penfluridol triggers autophagosome formation in non-small-cell lung cancer (NSCLC) cells. A549 and HCC827 cells were treated with penfluridol at the indicated concentrations for 24 h (**a**), or treated with penfluridol (10 μM) for indicated time points (**b**), and light chain 3 (LC3) conversion (LC3-I to LC3-II) was detected by a western blot analysis. Quantitative results of LC3B-II proteins were normalized to β-actin levels. **c** A549 cells were treated with penfluridol (5 µM) or chloroquine (20 μM) for 18 h. Cells were fixed and immunolabeled with an anti-LC3 antibody and revealed with an AlexaFluor488-conjugated secondary antibody. Nuclei were counterstained with DAPI (blue). Autophagosomes are indicated by the arrows. Original magnification, ×400. **d** Treatment of A549 or HCC827 cells with penfluridol (5 or 10 μM) for 12 h to detect other indicators of autophagosome formation including Atg5, Beclin-1, and p62 by a western blot analysis. Quantitative results of these proteins were normalized to β-actin levels. **e** Detection of acidic vesicular organelles (AVOs) by acridine orange staining after 7.5 μM penfluridol treatment for 6 h and analysis under a fluorescence microscope. The same cells were serum-starved for 48 h as an autophagy positive control. **f** A549 cells were incubated with penfluridol (5 μM), chloroquine (50 μM), or rapamycin (200 nM) for 18 h, followed by staining with LysoTracker Red (red) and immunolabeling with an anti-LC3 antibody (green). Nuclei were counterstained with DAPI (blue). The red puncta that overlayed with the green puncta (merged as yellow) were thus indicators of autolysosomes induced by rapamycin, whereas the solely green puncta were indicative of autophagosomes that are not fused with acidic lysosomes induced by penfluridol and chloroquine

Since we did not observe significant apoptosis after penfluridol treatment in NSCLC cells, we next investigated the time- and concentration-dependent effects of penfluridol on autophagy in NSCLC cells. The LC3 conversion (LC3-I to LC3-II), as a specific indicator of autophagosome formation, was induced by penfluridol in concentration-dependent (Fig. 3a) and time-dependent (Fig. 3b) manners in A549 and HCC827 cells. Notably, LC3B-II expression increased at as early as 6 h of treatment with penfluridol, indicating that penfluridol induced autophagosome formation from the early stage of treatment. Moreover, autophagosome formation induced by penfluridol in A549 cells was also confirmed by LC3B immunofluorescence. The redistribution of LC3 (Alexa Fluor 488, green) from the cytosol to autophagosomes indicates the formation of autophagosomes and CQ was used as a positive control for autophagosomes induction (Fig. 3c). In addition to LC3, other autophagosome markers such as Atg5 and Beclin-1 were also upregulated after penfluridol treatment (Fig. 3d). Autophagy is divided into normal flux and block in flux (autophagosomal accumulation)^31^. We next measured the effect of penfluridol on autophagic flux by detecting the level of p62, which was implicated in autophagic cargo recognition and reported to be incorporated into complete autophagosomes and to be degraded by autolysosomes^31^. Western blot results revealed that p62 levels increased in penfluridol-treated A549 and HCC827 cells (Fig. 3d), suggesting that penfluridol possibly inducing accumulation of autophagosomes reflects inhibition of their degradation in NSCLC cells. We further detected AVOs (by red fluorescence), which can be detected in autolysosomes by AO staining. Serum-starved NSCLC cells were used as a positive control for cells undergoing normal flux autophagy (Fig. 3e, lower panel). In contrast to serum-starved NSCLC cells, penfluridol treatment of NSCLC cells did not induce an increase in AVOs (Fig. 3e, middle panel). Moreover, we further observed that penfluridol can prevent the fusion of autophagosome with lysosome in NSCLC cells and rapamycin and CQ were respectively used as positive and negative control for autolysosomes induction (Fig. 3f). Taken together, these results demonstrated that penfluridol treatment resulted in blocking autophagic flux and inducing autophagosome accumulation, but not autophagosome–lysosome fusion in NSCLC cells.

### Penfluridol-mediated growth inhibition of human NSCLC cells is dependent on autophagosome accumulation

**Fig. 4:**
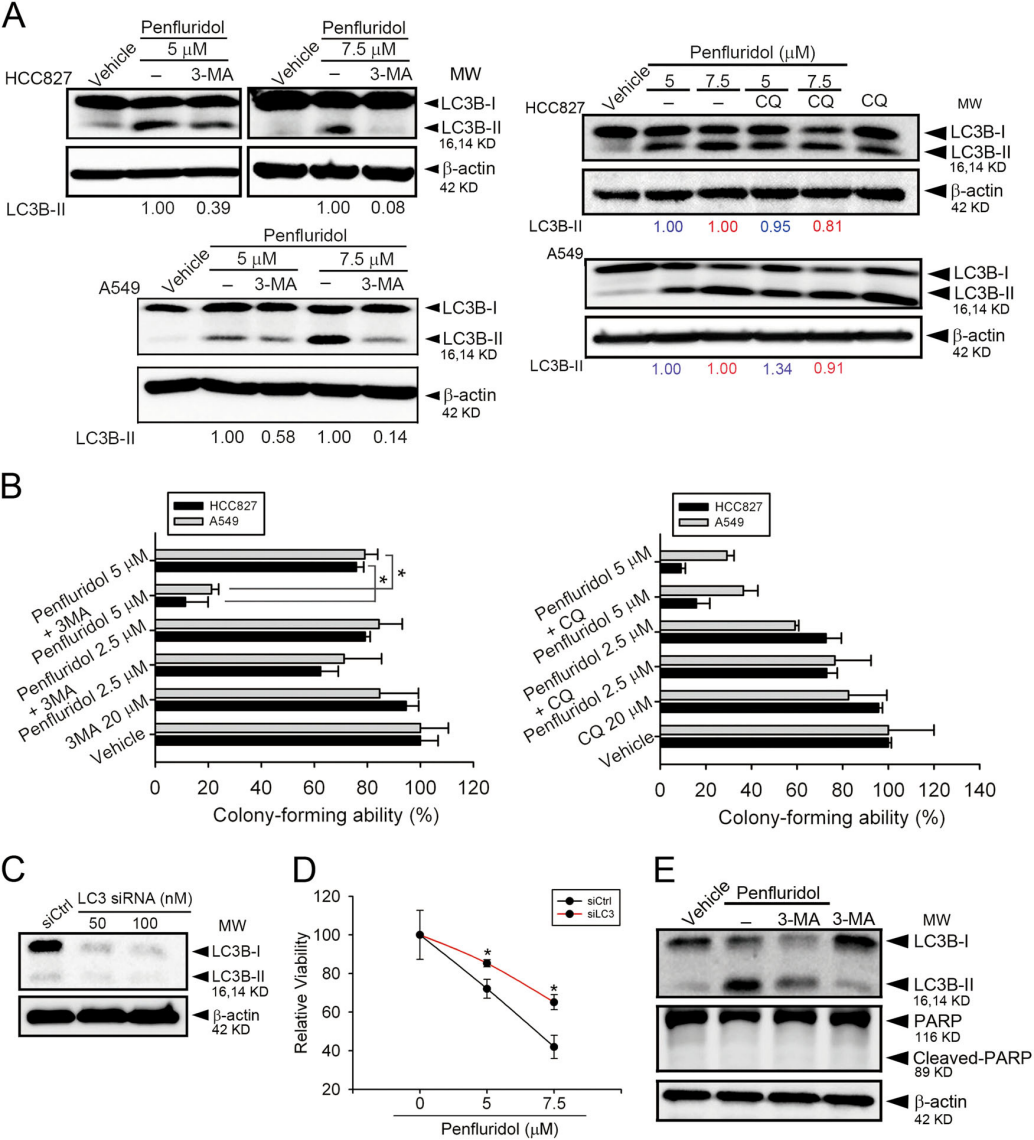
Autophagosome accumulation is critical for the loss of viability caused by penfluridol in non-small-cell lung cancer (NSCLC) cells. **a**, **b** A549 and HCC827 cells were pretreated with 3-methylamphetamine (3-MA) (20 μM) or chloroquine (CQ) (20 μM) for 1 h, followed by penfluridol (2.5, 5, or 7.5 μM) treatment for 24 h. Light chain 3 (LC3) conversion in both cell lines was detected by a western blot analysis (**a**). Quantitative results of LC3B-II proteins were normalized to β-actin levels. The death-inducing effects of penfluridol, penfluridol + 3-MA, and penfluridol + CQ on cells were determined by counting the colonies formed (**b**). Data are presented as the mean ± SD of three independent experiments. *p < 0.05, compared to the penfluridol + 3-MA group. **c**, **d** A549 cells were transiently transfected with LC3-specific siRNA or control siRNA and subjected to an MTS assay. Knockdown efficiency of LC3 siRNA was confirmed by a western blot analysis (**c**). LC3-specific siRNA reversed the penfluridol-induced decrease of cell proliferation in A549 cells (**d**). Data are presented as the mean ± SD of three independent experiments. *p < 0.05, compared to the siCtrl-transfected group. **e** A549 cells were pretreated with 3-MA for 1 h, followed by 7.5 μM penfluridol treatment for 24 h. The LC3 conversion and poly(ADP ribosome) peroxidase (PARP) cleavage were detected by a western blot analysis

To further confirm the role of penfluridol in blocking autophagic flux in NSCLC cells, inhibitors of autophagosome formation (the early autophagy inhibitor, 3-MA) and autophagosome–lysosome fusion (the late autophagy inhibitor, CQ) were used. We found that 3-MA dramatically reversed the penfluridol-mediated increase in LC3B-II expression (Fig. 4a, left panel), but CQ treatment had no significant effect on LC3B-II levels in penfluridol-treated NSCLC cells (Fig. 4a, right panel). In addition, we observed that 3-MA, but not CQ, dominantly reversed penfluridol-induced inhibition of colony formation in A549 and HCC827 cells (Fig. 4b). Furthermore, we used siRNA against LC3 to determine penfluridol-induced cell growth inhibition in A549 cells. Figure 4c shows dramatic decreases in LC3 expression in cells transfected with LC3-specific siRNA. Silencing of LC3 significantly rescued penfluridol-mediated growth inhibition (Fig. 4d). These results suggested that penfluridol may confer cytotoxicity mainly through inducing autophagosome accumulation, but not complete autophagic flux in NSCLC cells. There is much evidence that autophagy and apoptosis are interdependent, and activation of these two death machineries most often occurs simultaneously^32^. However, pretreatment of A549 cells with 3-MA dramatically reversed penfluridol-induced LC3B-II production, but had no effect on PARP cleavage (Fig. 4e), indicating that autophagosome accumulation-mediated cell death is independent of apoptosis.

### Anticancer effects of penfluridol via inducing autophagosome accumulation in an A549 orthotopic graft model

**Fig. 5:**
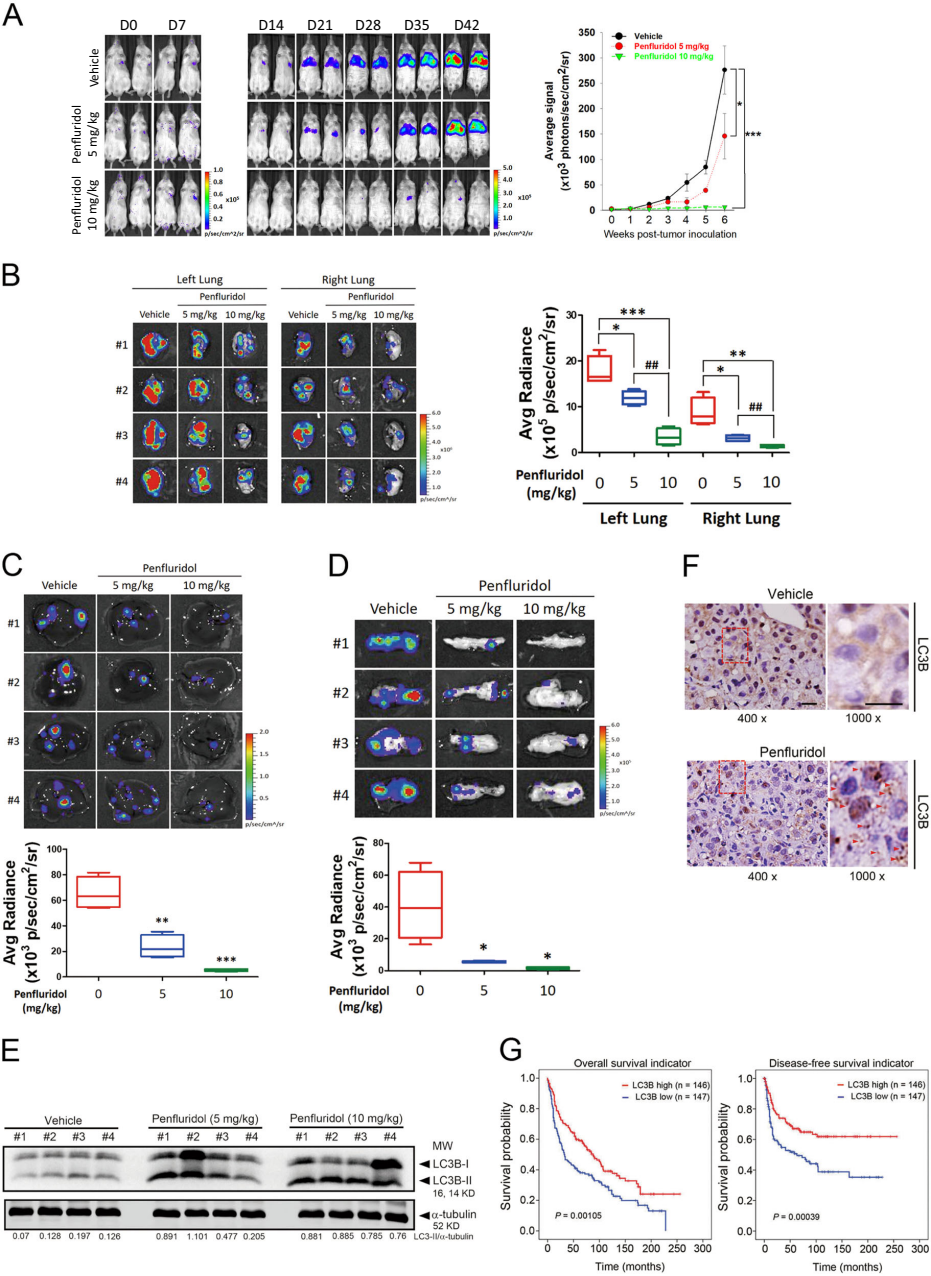
Anticancer effects of penfluridol via inducing autophagosome accumulation in an A549 orthotopic graft model. Luciferase-tagged A549 cells were orthotopically implanted into the left lateral thorax of NOD/SCID mice. After tumor cell injection for 1 week, mice were orally administered penfluridol (5 or 10 mg/kg) or vehicle 5 days/week for 5 consecutive weeks. **a** Left panel: xenogen IVIS spectrum bioluminescence imaging of orthotopic lung tumor growth. Right panel: quantitative analysis of Xenogen imaging signal intensity (photons/s/cm^2^/steradian) at the indicated time points. *p < 0.05; ***p < 0.001 compared with the vehicle control group. **b** Left panel: cancer metastasis from the left lung to the right lung was imaged by bioluminescence. Right panel: signal intensities from primary tumors (left lung) and metastatic tumors (right lung) were bioluminescently captured at the end of the study, with the mean signal for each group indicated. *p < 0.05, **p < 0.01, ***p < 0.001 compared with the control group. ^##^*p* < 0.01 compared with the 5 mg/kg penfluridol-treated group. Upper panel: cancer distal metastasis, including liver (**c**) and pancreas (**d**) metastasis, was imaged with bioluminescence at the end of the study. Lower panel: liver (**c**) and pancreas (**d**) metastatic signal intensities were bioluminescently captured with the mean signal for each group indicated. *p < 0.05, **p < 0.01, ***p < 0.001 compared with the control group. A549 orthotopic tumors treated with vehicle or penfluridol were isolated to detect light chain 3 (LC3) conversion by a western blot analysis (**e**) and autophagosome formation by IHC staining of LC3 (**f**). Quantitative results of LC3B-II proteins were normalized to α-tubulin levels. LC3 dot-like staining is indicated by an arrow. (Left: original magnification ×400; Right: original magnification ×1000). **g** Kaplan–Meier analysis of *LC3B* gene expressions in lung cancer tissues (GSE30219)

To further test the efficacy of penfluridol in inhibiting the growth and metastasis of NSCLC cells in vivo, we established an orthotopic lung tumor-bearing model by transplanting A549-Luc cells into the left lung of NOD-SCID mice, and we allowed them to become established for 7 days before initiating treatment. The experimental group of mice was treated with 5 or 10 mg/kg penfluridol by oral gavage 5 days/week, whereas mice in control group received vehicle only. The effects of penfluridol administration on tumor growth and metastasis were monitored by bioluminescence imaging. From in vivo photon emission detection, we found that penfluridol treatment concentration-dependently suppressed tumor growth compared with the vehicle control group (Fig. 5a). Comparable with in vivo data, ex vivo imaging of the lungs from sacrificed mice revealed lower photon intensities in penfluridol-treated mice compared with vehicle-treated mice, in both orthotopic tumors (in the left lung) and metastatic tumors (in the right lung) (Fig. 5b). In addition to lung tissues, distant metastases of cells to the liver (Fig. 5c) and pancreas (Fig. 5d) were significantly suppressed by penfluridol treatment. A portion of primary lung tumor tissues was snap-frozen for western blotting, whereas another part was fixed for IHC staining. Consistent with our in vitro findings, these results showed that tumors from the penfluridol-treated group exhibited more autophagosome accumulation as evidenced by an increase in LC3B-II expression, compared with the vehicle control group (Fig. 5e). From data of IHC staining, we also observed that dot-like staining of LC3B had also increased in tumors isolated from penfluridol-treated mice (Fig. 5f). Moreover, we analyzed the prognostic significance of LC3B expression using gene expression data obtained from the publicly available Gene Expression Omnibus database (GSE30219). Results from a Kaplan–Meier plot showed that patients with lung tumors exhibiting high expression levels of LC3B had significantly longer overall (*P* = 0.00105) and disease-free survival (*P* = 0.00039) times compared with patients with tumors exhibiting low expression levels of LC3B (Fig. 5g). These results clearly demonstrated that autophagosome accumulation may play a critical role in the penfluridol-mediated suppressive effect for NSCLC progression and may be associated with the good prognosis of patients with lung cancer.

### The UPR and p38 MAPK signaling pathways are involved in penfluridol-induced autophagosome accumulation and autophagosome accumulation-mediated cell death is mainly attributed to ATP energy loss

**Fig. 6:**
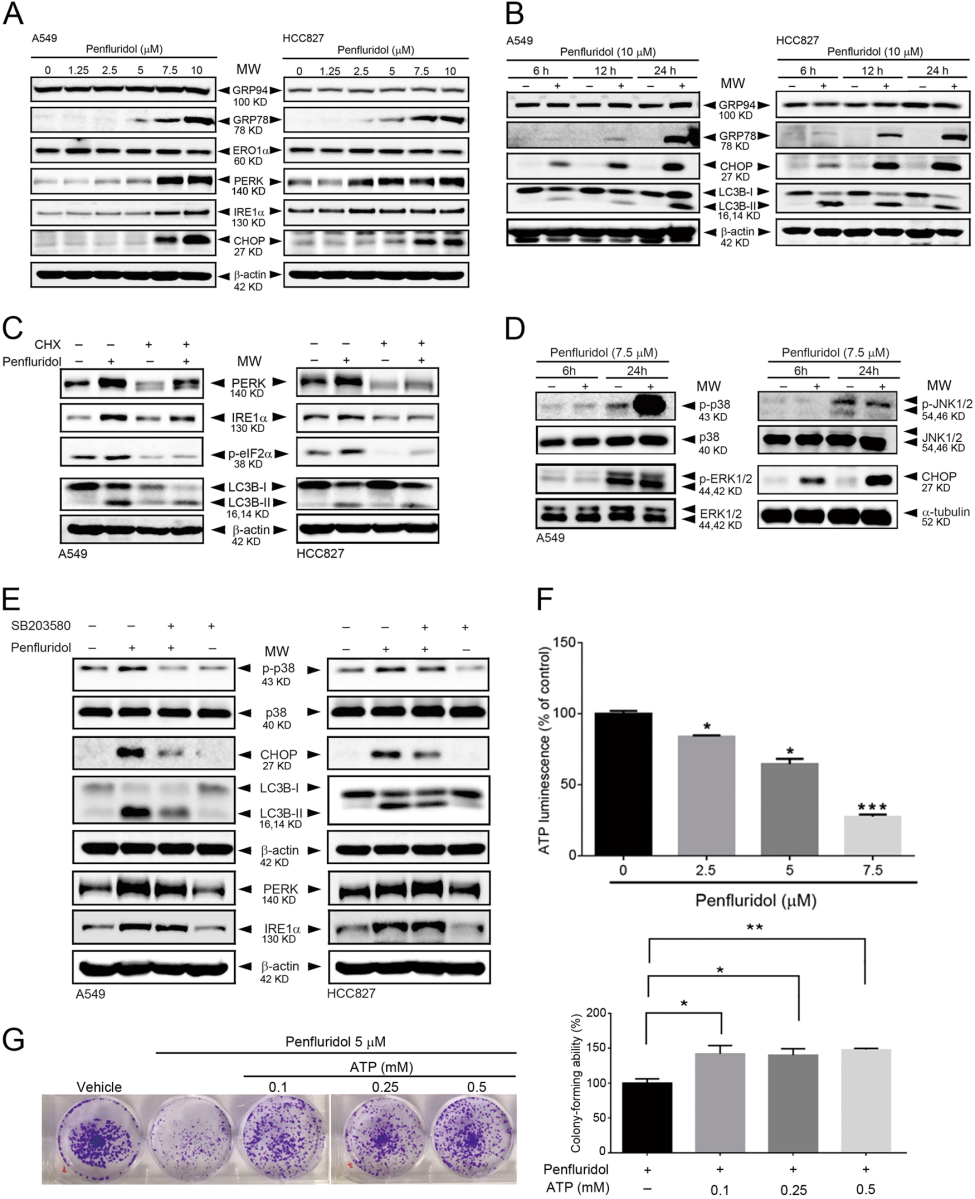
The unfolded protein response (UPR) and p38 mitogen-activated protein kinase (MAPK) signaling pathways are involved in penfluridol-induced autophagosome accumulation and autophagosome accumulation-mediated cell death is mainly attributed to ATP energy loss. A549 and HCC827 cells were treated with various concentrations of penfluridol for 24 h (**a**) or 10 μM penfluridol for indicated time points (**b**), and UPR signals, GRP94, GRP78, ERO1α, PERK, IRE1α, and CHOP, were detected by a western blot analysis. **c** A549 and HCC827 cells were pretreated with or without cycloheximide (35 μM) for 1 h, followed by penfluridol (7.5 μM) treatment for 24 h. Light chain 3 (LC3) conversion and UPR signals in both cells were detected by a western blot analysis. **d** Phosphorylation levels of p38 MAPK, extracellular signal-regulated kinase 1/2 (ERK1/2), and c-Jun N-terminal kinase 1/2 (JNK1/2) were assessed using a western blot analysis after treatment of A549 cells with 7.5 μM penfluridol for the indicated time points. **e** A549 and HCC827 cells were pretreated with or without SB203580 (1 μM) for 1 h followed by penfluridol (7.5 μM) treatment for an additional 24 h. LC3 conversion and UPR signals in both cell lines were detected by a western blot analysis. **f** A549 cells were treated with indicated concentrations of penfluridol for 24 h and harvested for the detection of intracellular ATP by a Luminescent ATP Detection Assay Kit ab113849 from Abcam (Cambridge, MA). *p < 0.05, ***p < 0.001 compared with the control group. **g** A549 cells were co-treated with or without ATP (0.1, 0.25, or 0.5 mM) and penfluridol (5 μM) for 24 h; then, the death-inducing effects of penfluridol on cells were determined by counting the colonies formed. Left: representative photomicrographs. Right: data are presented as the mean ± SD of three independent experiments. *p < 0.05, **p < 0.01 compared with the penfluridol treatment only group

When misfolded proteins accumulate in the ER to activate the UPR, a preautophagosomal structure is assembled and transported to vacuoles to turn on autophagy^14^. Hence, we next investigated whether penfluridol induces ER stress in A549 and HCC827 cells. After screening the UPR signaling pathways such as GRP94, GRP78/BIP, ERO1α, PERK, IRE1α, and CHOP, in penfluridol-treated NSCLC cells, we found that GRP78, PERK, IRE1α, and CHOP as well as LC3B-II were concentration- (Fig. 6a) or time-dependent (Fig. 6b) upregulated after penfluridol treatment. To further investigate whether ER stress induced by penfluridol leads to autophagosome accumulation, we pretreated NSCLC cells with the ER stress inhibitor, cycloheximide, followed by treatment of cells with penfluridol. We found that penfluridol-induced increases in UPR signals and LC3B-II were all significantly reversed by cycloheximide (Fig. 6c), suggesting that the ER stress-mediated UPR is involved in penfluridol-induced autophagosome accumulation in human NSCLC cells. The cross talk between mitogen-activated protein kinases (MAPKs) and UPR was reported in cancers^33^. We therefore examined whether the MAPK pathways were involved in the penfluridol-mediated UPR and autophagosome accumulation in NSCLC cells. Our results showed that treatment of A549 cells with penfluridol for different time points (6 and 24 h) induced p38 MAPK activation, as well as CHOP expression, but had no significant effect on JNK1/2 or ERK1/2 activation (Fig. 6d). The concentration-dependent effect of penfluridol on p38 MAPK activation was also observed in HCC827 cells (Fig. S3). Moreover, treatment of A549 or HCC827 cells with the p38 MAPK inhibitor, SB203580, considerably reversed penfluridol-induced CHOP and LC3B-II expressions, but did not reverse penfluridol-induced PERK and IRE1α (Fig. 6e), suggesting that p38 MAPK participates in the penfluridol-induced UPR and autophagosomes accumulation in NSCLC cells. Excessive autophagosome accumulation was reported to deplete energy and thereby exert cell toxicity^7^. We therefore tested the effect of penfluridol on ATP energy level of NSCLC cells and found that a significant decrease in ATP levels with penfluridol treatment was observed in NSCLC cells (Fig. 6f). Moreover, replenishment of ATP to penfluridol-treated cell can significantly reverse penfluridol-induced growth inhibition of NSCLC cells (Fig. 6g), suggesting that autophagosome accumulation-mediated cell death is attributed to ATP energy loss.

## Discussion

NSCLCs are typically resistant against apoptosis induced by standard chemotherapy^34^. Autophagy has recently emerged as an important target in cancer^35^. In this study, we first demonstrated that penfluridol, a US Food and Drug Administration-approved antipsychotic drug, induced nonapoptotic cell death of NSCLCs through blocking autophagic flux and inducing autophagosome accumulation-mediated ATP energy depletion. The amount of autophagosomes in the cytosol can be increased by both increasing autophagosome synthesis and blocking lysosomal degradation at a later stage. Our present results showed that penfluridol induced an increase in autophagosome synthesis according to the induction of LC3B-II conversion by penfluridol. Moreover, the penfluridol-induced increase in the autophagy-specific substrate, p62, further indicated that penfluridol-induced accumulation of autophagosomes may occur through inhibiting their degradation by lysosomes. Actually, our results also demonstrated that penfluridol can prevent the fusion of autophagosome with lysosome in NSCLC cells. Multiple complexes appear to regulate autophagosome–lysosome fusion such as lysosomal-associated membrane proteins 1 (LAMP-1) and 2 (LAMP-2) and syntaxin (STX)−17. Although our present study indicated that penfluridol might suppress autophagosome–lysosome fusion, the effect of penfluridol on these regulators should be further investigated.

Recent studies indicated that disruption of the late stage of autophagy leads to excessive accumulation of autophagosomes and has the potential to turn autophagy into a destructive process^7,8^. Autophagy is an important source of energy, especially for high growth demands of cancer cells, and it can also help remove harmful products from the cancer microenvironment. According to this property, NSCLC often upregulate autophagy and can be more autophagy-dependent than most normal tissues^36^. Therefore, these cancers may be more susceptible to autophagosome accumulation-based toxicity. Actually, our study showed that NSCLC cells were more sensitive to penfluridol-induced cell death than were normal lung epithelial cells. Recently, combining chemotherapeutic drugs or EGFR tyrosine kinase inhibitors (TKIs) with autophagosome clearance inhibitors was proven to increase the treatment potency against NSCLC cells^37,38^. The combination of penfluridol and an EGFR TKI or chemotherapeutic drugs for treating NSCLC is worthy of further clinical evaluation.

As to the mechanisms underlying the penfluridol-induced autophagosome accumulation toxicity in NSCLC cells, recent studies indicated that impaired autophagic flux is associated with accumulation of misfolded proteins and ER stress^39^. The cross talk of autophagy and apoptosis, mostly in a sequence in which autophagy precedes apoptosis, was reported to be induced by ER stress^40^. Moreover, previous studies indicated that penfluridol-induced ER stress leads to autophagy and finally induces cell apoptosis in pancreatic cancer^25,27^. Our present study demonstrated that penfluridol treatment could induce ER stress-mediated UPR signaling pathways, which led to autophagosome accumulation in NSCLC cells. To our surprise, we observed that penfluridol did not induce apoptosis of NSCLC cells, and inhibition of penfluridol-mediated LC3-II conversion by 3-MA also did not affect the apoptotic effect on cells, suggesting that ER stress-induced autophagosome accumulation and cell death by penfluridol are independent of apoptosis. This result is similar to that of a previous report, which indicated that accumulation of autophagosomes causes loss of cell viability independent of apoptosis^7^. In addition to misfolded proteins, autophagy is also important in reducing intracellular ROS through elimination of damaged mitochondria, the major source of ROS^41^. Accumulation of autophagosomes was recently proven to cause an increase in ROS in HEK293 cells^7^. The contrasting roles of low and high ROS concentrations in tumor progression and drug resistance of NSCLC were defined. High ROS levels were shown to have toxic effects on cancer cells, by triggering several signal transduction pathways, such as p38/JNK MAPK signaling, and then inducing cell cycle arrest, cell death, and overcoming TKI resistance^42^. Indeed, our present data showed that upregulation of ROS was induced by penfluridol in NSCLC cells (Fig. S4). In breast cancer, penfluridol was reported to induce ROS-mediated cell growth inhibition and apoptosis via significantly downregulating specificity protein (Sp) 1, Sp3, and Sp4^24^. In contrast, our results showed that pretreatment of A549 cells with ROS scavenger, GSH, or NAC, cannot reverse penfluridol-induced increase of LC3B-II expression (Fig. S5A) and inhibition of cell viability and colony formation (Fig. S5C, D), suggesting ROS induction is not a key driver of penfluridol-induced autophagosome accumulation and cell growth inhibition in NSCLC cells. Moreover, treatment of penfluridol with A549 cells did not inhibit or slightly enhanced the expression of Sp1, Sp3, and Sp4 (Fig. S6), suggesting the underlying mechanisms involved in the anticancer action of penfluridol in NSCLC and breast cancer are different. In addition to cell death, cell cycle G_0_/G_1_ arrest and its related regulator, p21 (Fig. S7), were also induced by penfluridol in NSCLC cells, but the role of ROS in penfluridol-induced cell cycle arrest should be further determined in the future.

In addition to eliminating intracellular toxins, autophagy also plays an important role in supplying energy for cancer cells via the degradation and recycling of unnecessary materials^36^. Excessive autophagosome accumulation was reported to deplete energy and thereby exert cell toxicity^7^. Actually, a significant decrease in ATP levels with penfluridol treatment was observed in NSCLC cells. Moreover, replenishment of ATP to penfluridol-treated cell can significantly reverse penfluridol-induced UPR signals, LC3 conversion (Fig. S5A, B), and growth inhibition of NSCLC cells, suggesting that autophagosome accumulation-mediated cell death is attributed to ATP energy loss. Glucose deprivation-mediated ATP level low has been reported to activate UPR-dependent cell death, especially in cancer cells^43^, suggesting penfluridol-induced ATP loss might positively feedback regulate UPR in NSCLC cells.

A recent study showed that MAPK signaling pathways have a role in the response to ER stress. For example, p38 MAPK and JNK are activated by ER stress and form part of the UPR^33^. Hence, we evaluated the intracellular cross talk between the MAPK pathways in the penfluridol-induced UPR. We found that penfluridol can simultaneously induce activation of p38 and expression of the transcription factor, CHOP, even 24 h after treatment. Long-term activation of p38 and overexpression of CHOP were reported to promote cell cycle arrest through induction of p21 or p27^44,45^. Moreover, we also observed that the p38-specific inhibitor reversed expression of CHOP and conversion of LC3B-II induced by penfluridol, but had no effect on penfluridol-induced RERK and IRE1α expressions in both A549 and HCC827 cells. These results suggest that penfluridol might induce the p38-CHOP pathway to induce cell cycle arrest and autophagosome accumulation-mediated cell death in NSCLC cells. Recently, ER stress-mediated p38 activation was reported to phosphorylate CHOP on serine 79 and serine 81 to promote its transcriptional activity and induce cell death^33^. Taken together, penfluridol might trigger the p38 MAPK-mediated CHOP phosphorylation pathway to induce cell death in NSCLC cells, but this issue should be further investigated in our future work.

**Fig. 7:**
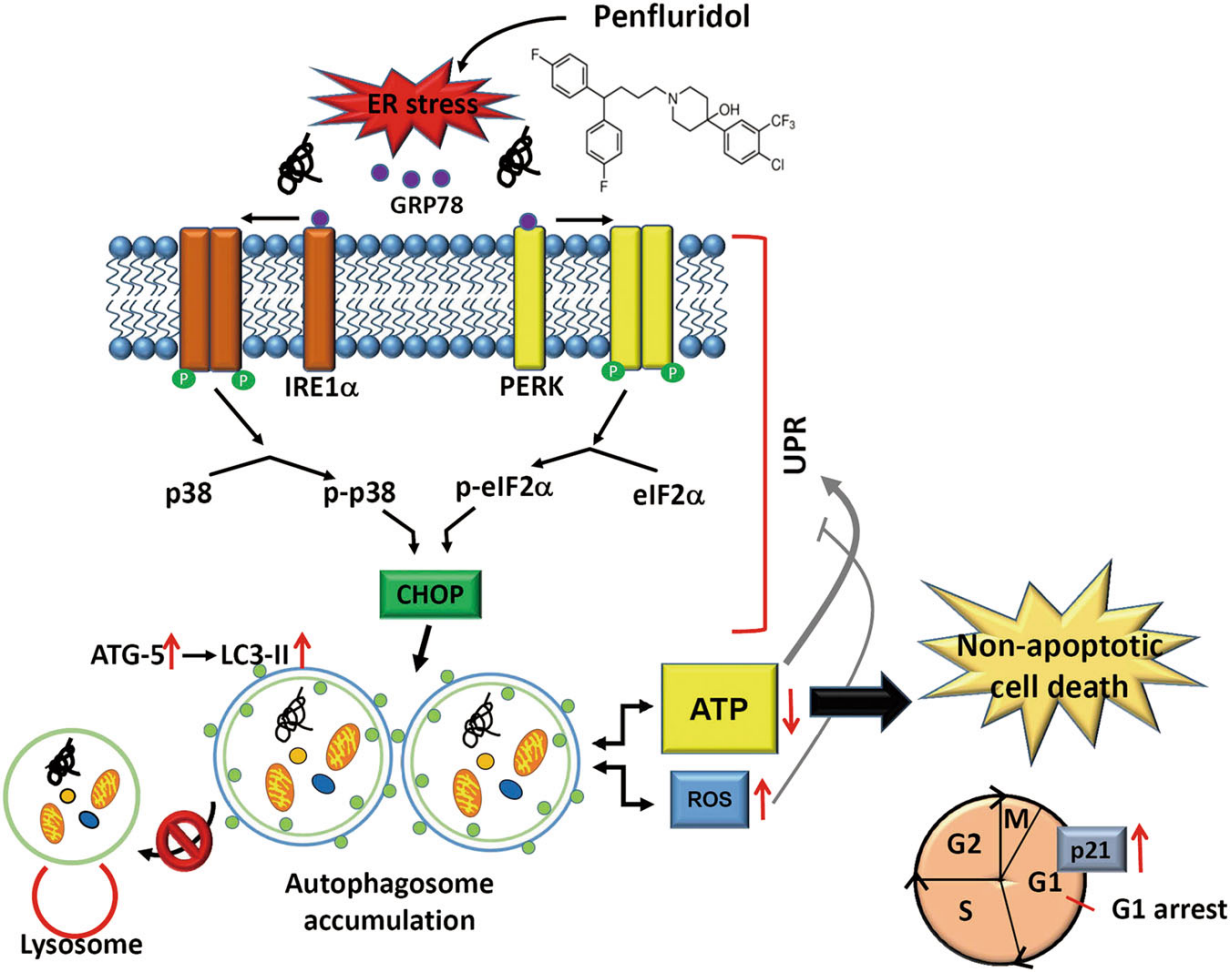
A working model shows the molecular mechanisms underlying the ability of penfluridol to suppress growth of NSCLC cells. The anticancer activity of penfluridol on NSCLC cells was attributed to induction of ER stress leading to UPR-mediated autophagosome accumulation and subsequently induction of intracellular ATP depletion. p38 MAPK was also involved in part of the penfluridol-mediated UPR and autophagosome accumulation. Finally nonapoptotic cell death and G_1_ phase cell-cycle arrest were induced by penfluridol to suppress growth of NSCLC cells

In summary, the present data fist demonstrated that penfluridol-induced UPR signaling leads to autophagosome accumulation-mediated ATP energy loss, resulting in lung tumor growth suppression via nonapoptotic cell death. p38 MAPK was proven to be involved in part of the penfluridol-mediated UPR and autophagosome accumulation; the mechanism is schematically illustrated in Fig. 7. Our present findings strongly support penfluridol as a repurposed drug for treating NSCLC, especially for the cancer types that are resistant to chemotherapy or EGFR TKIs.

## Supplementary information

Supplementary data
