# Supplementary material for: Autophagosome accumulation-mediated ATP energy deprivation induced by penfluridol triggers nonapoptotic cell death of lung cancer via activating unfolded protein response
Source: Cell Death Dis. 2019 Jul 15;10(8):538. doi: 10.1038/s41419-019-1785-9 (PMC6629704; doi:10.1038/s41419-019-1785-9)
Supplement: Supplementary file 1 — Supplementary data [file 41419_2019_1785_MOESM1_ESM.docx]

**Supplemental Information**

**Title:**

**Autophagosome accumulation-mediated ATP energy deprivation induced by penfluridol triggers non-apoptotic cell death of lung cancer via activating unfolded protein response**

Wen-Yueh Hung, Jer-Hwa Chang, Yu Cheng, Guo-ZhouCheng, Hsiang-Ching Huang, Michael Hsiao, Chi-Li Chung, Wei-Jiunn Lee, and Ming-Hsien Chien

Correspondence to: Dr. Wei-Jiunn Lee (E-mail: lwj5905@gmail.com) and Dr. Ming-Hsien Chien (E-mail: mhchien1976@gmail.com)

**Figure Legends**

**
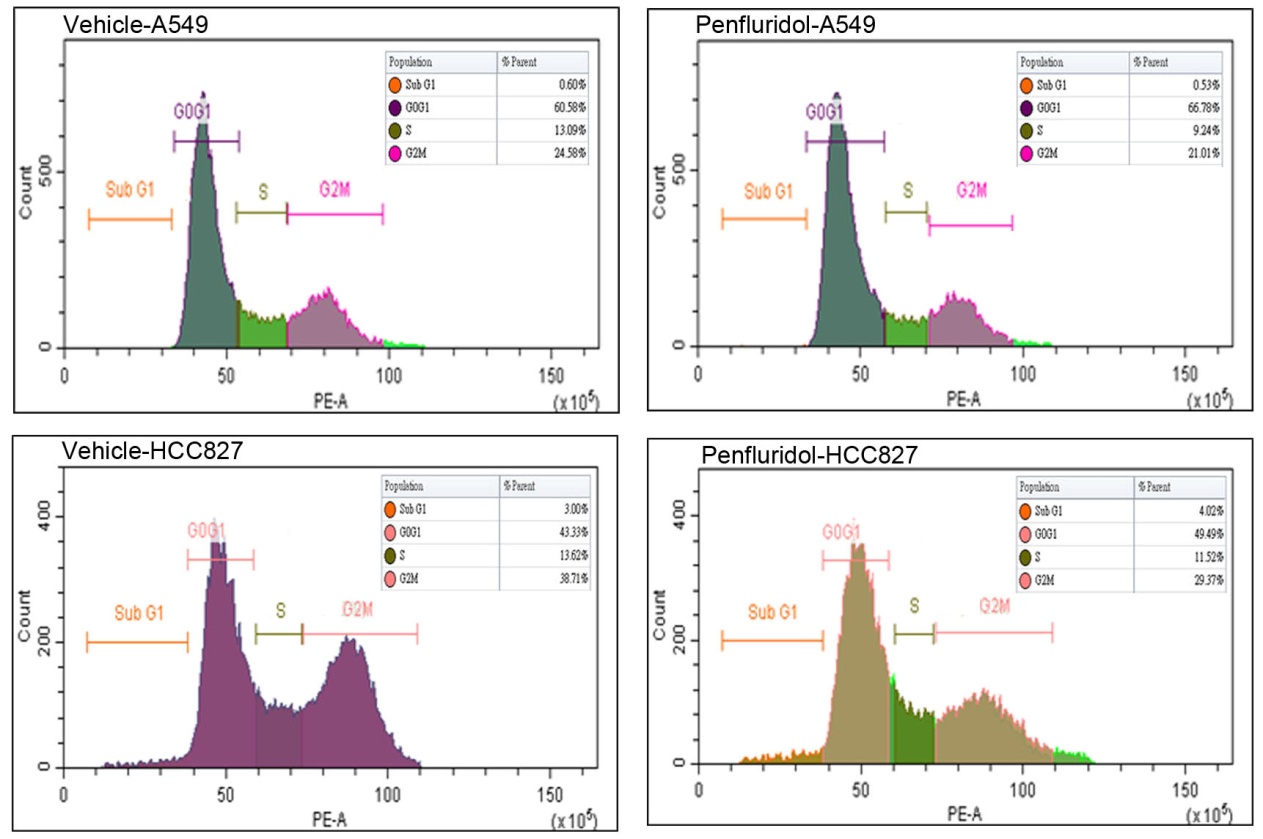
**

**Figure S1.** Penfluridol induces an increase in the cell population in the G_0_/G_1_ phase, but not in the sub-G_1_ phase. Treatment of A549 and HCC827 cells with penfluridol (5 µM) for 24 h. The cell-cycle phase distribution and cell death in the sub-G_1_ phase were analyzed by FACS after propidium iodide (PI) staining. Data are shown as the cell-cycle distribution profile by FACS, and percentage distributions of cells in the sub-G_1_, G_0_/G_1_, S, and G_2_/M phases are inserted in the graphs.


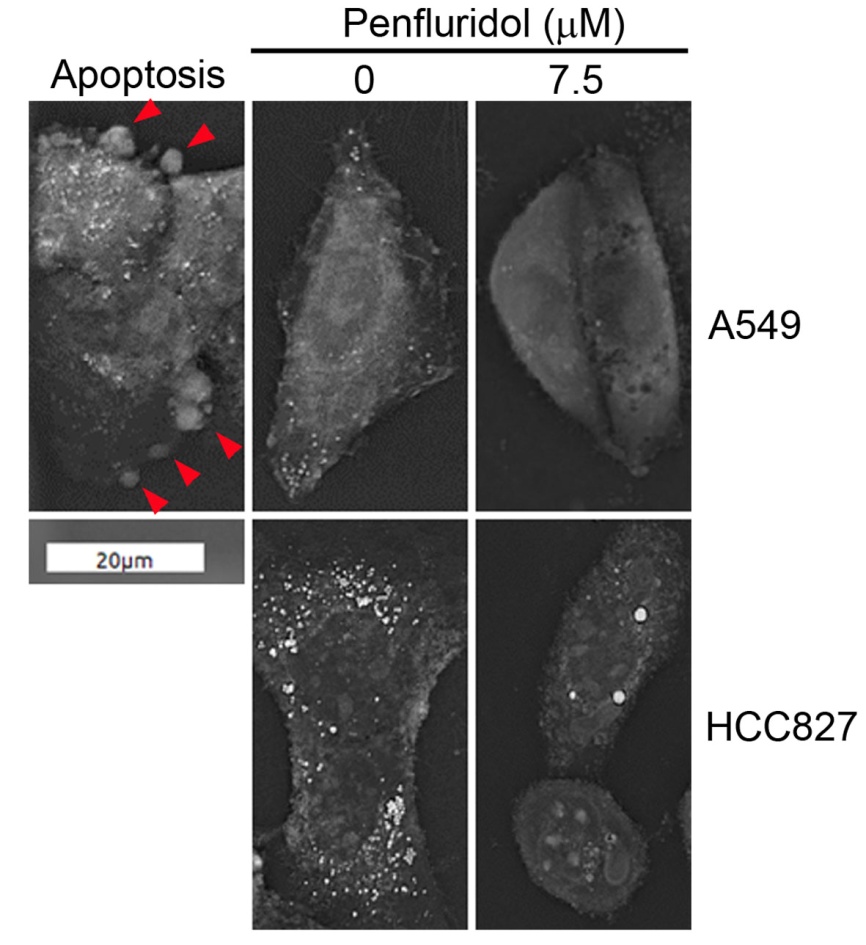


**Figure S2.** Effects of penfluridol on changes of apoptotic features in A549 and HCC827 cells. Under holotomographic microscopy, the formation of apoptotic bodies (red arrow indicated) was evaluated in A549 and HCC827 cells treated with penfluridol (7.5 μM) for 24 h.

**
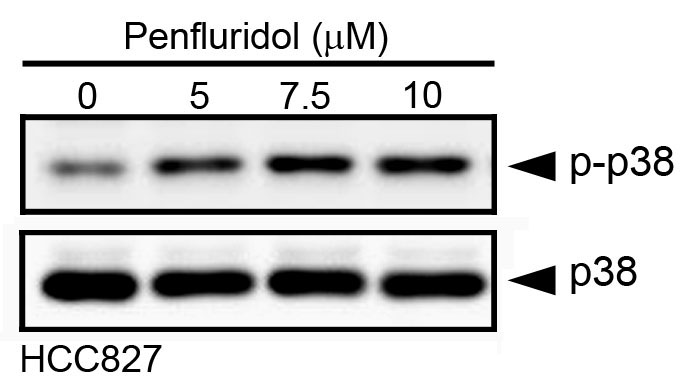
**

**Figure S3.** Penfluridol induces phosphorylation of p38 mitogen-activated protein kinase (MAPK) in a concentration-dependent manner in HCC827 cells. Cells were treated with indicated concentrations of penfluridol for 24 h and harvested for the detection of p21 and p27 expressions by a Western blot analysis.


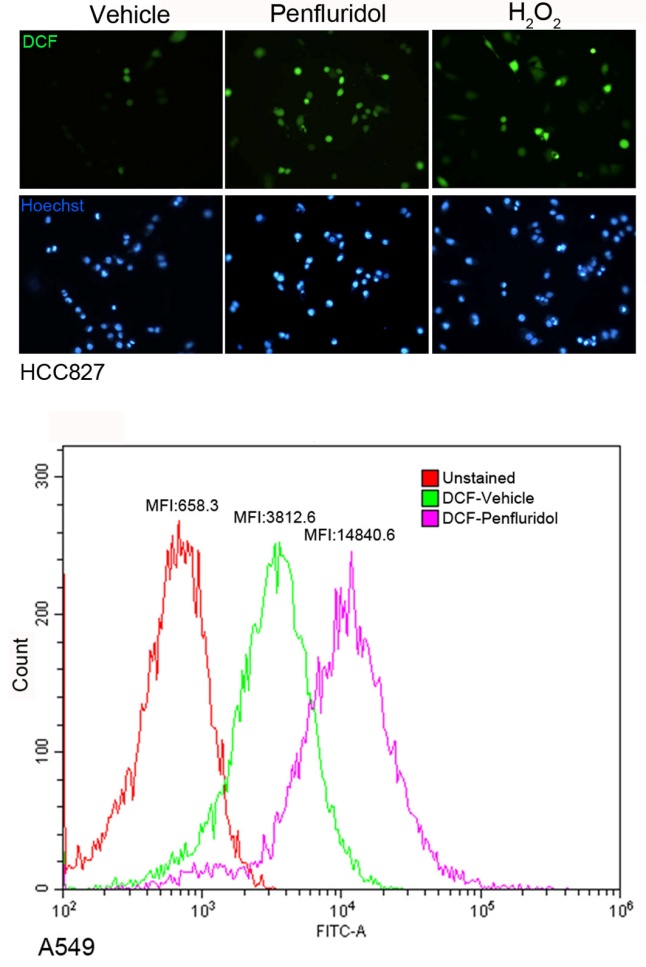


**Figure S4.** Oxidative stress was induced by penfluridol in A549 and HCC827 cells. Cells were treated with 7.5 μM penfluridol or 100μM H_2_O_2_ for 6 h and stained with DCF-DA. Upper panel, the fluorescence image of DCF-stained HCC827 cells was taken with a fluorescence microscope. Lower panel, the fluorescence intensity of DCF-stained A549 cells was quantified by FACS. Mean fluorescence intensity (MFI) was inserted in the graphs.

**
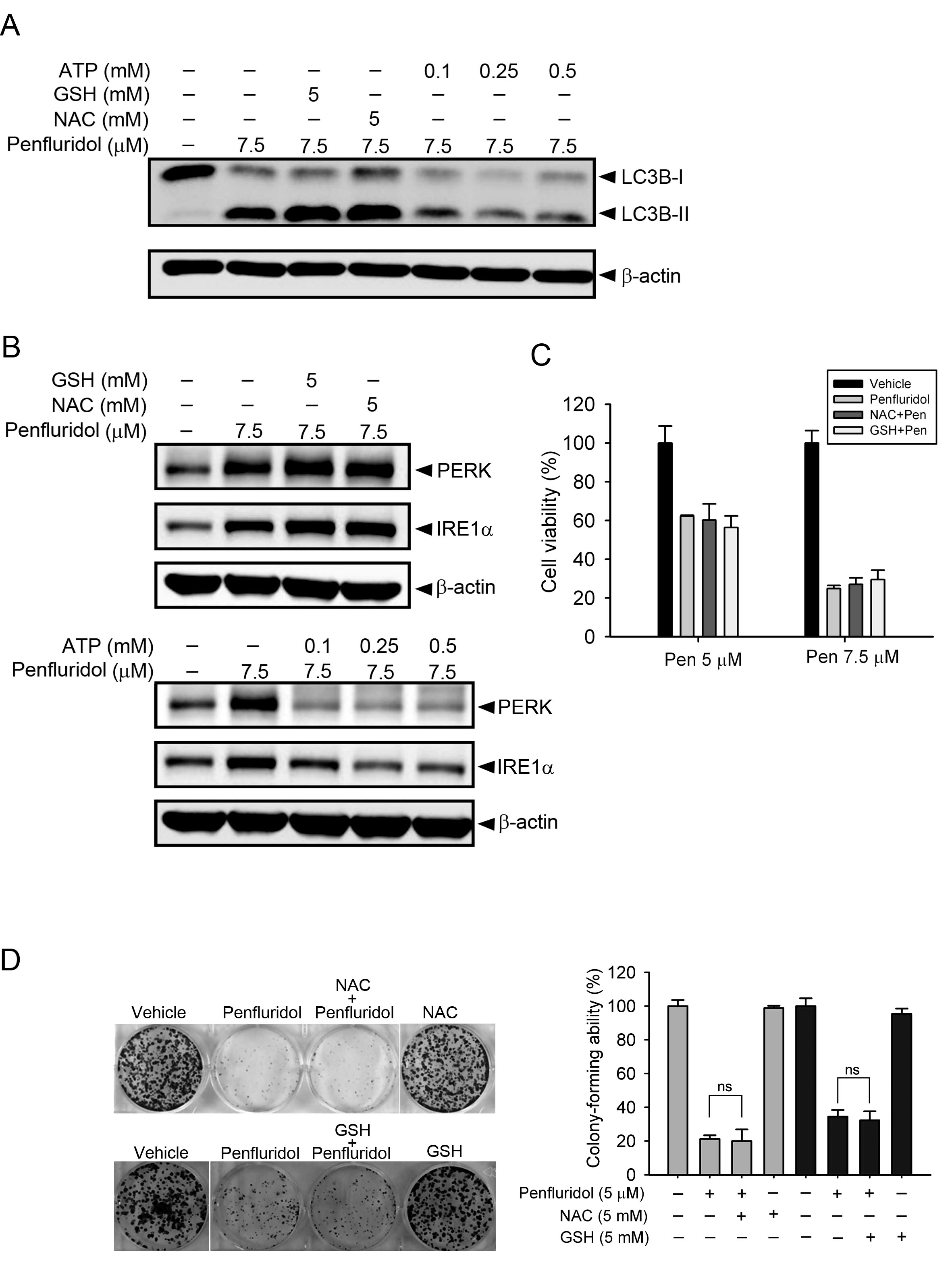
**

**Figure S5.** Inhibition of oxidative stress production cannot reverse the penfluridol-induced unfolded protein response (UPR), light chain 3 (LC3) conversion, and cell growth inhibition in A549 cells. (A and B) A549 cells were pretreated with or without NAC or GSH (5 mM) for 1 h followed by penfluridol (7.5 μM) treatment for an additional 24 h. LC3 conversion (A) and UPR signals (B) were detected by a Western blot analysis. (C and D) A549 cells were pretreated with or without NAC or GSH (5 mM) for 1 h followed by penfluridol (5 or 7.5 μM) treatment for an additional 24 h. The death-inducing effects of penfluridol on cells were determined by an MTS assay (C) or counting the colonies formed (D). ns: not significant


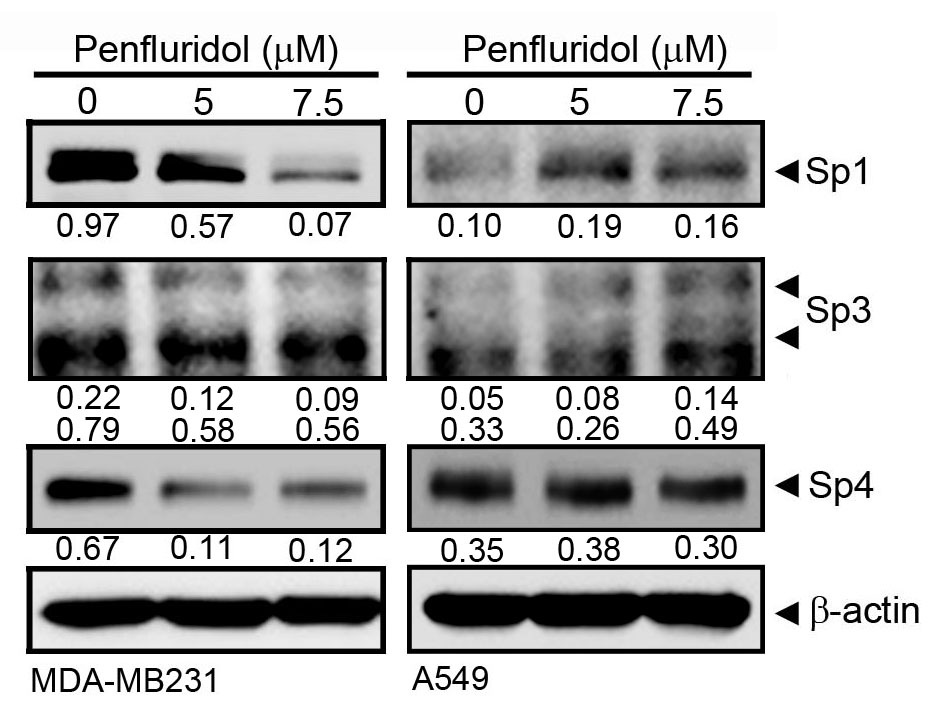


**Figure S6.** Effect of penfluridol on expression of specificity protein (Sp) 1, Sp3, and Sp4 in A549 cells. Expression of Sp1, Sp3, and Sp4 were evaluated by a Western blot analysis after treatment of A549 cells with 5 or 7.5 μM penfluridol for 24 h. MDA-MB231 cells were used as a positive control for penfluridol-induced downregulation of Sp1, Sp3, and Sp4. Quantitative results of Sp proteins were normalized to β-actin levels.


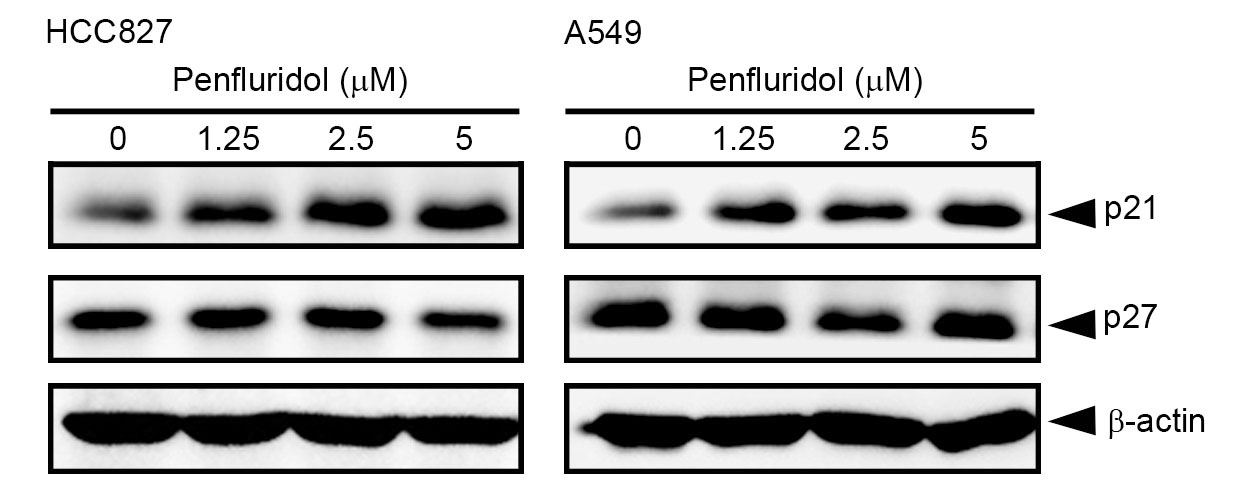


**Figure S7.** p21 but not p27 proteins were induced by penfluridol in A549 and HCC827 cells. Cells were treated with indicated concentrations of penfluridol for 24 h and harvested for the detection of p21 and p27 expression by a Western blot analysis. Quantitative results of p21 and p27 proteins were normalized to β-actin levels.
